# Therapeutic Potential of Polyphenols in Targeting Th17/Treg Balance for Intestinal Barrier Maintenance

**DOI:** 10.1002/fsn3.70400

**Published:** 2025-06-07

**Authors:** Jiaojiao Fu, Zhenghua Zhao, Da Zhou, Sijing Liu

**Affiliations:** ^1^ Department of Clinical Laboratory Chengdu Sixth People's Hospital Chengdu P. R. China; ^2^ College of Medical Technology Chengdu University of Traditional Chinese Medicine Chengdu P. R. China

**Keywords:** intestinal barrier, polyphenol, Th17/Treg balance

## Abstract

The integrity of the intestinal barrier is essential for overall health. Disruption of tight junction (TJs) proteins negatively affects intestinal barrier function, leading to immune imbalances and causing a variety of pathological conditions that are closely associated with the pathogenesis of multiple diseases. Th17 and Treg cells are two distinct phenotypes of CD4^+^ T cells with opposing functions. The balance established between these two subpopulations is essential to prevent the development of disease. Emerging evidence suggests that the Th17/Treg balance is an important potential target for improving the intestinal barrier. However, there is still a lack of effective drugs for the treatment of intestinal barrier dysfunction or disorders. Polyphenols, natural compounds found in plants, have gained attention as potential therapies. Research shows that polyphenols can regulate the Th17/Treg balance by influencing key molecular pathways, such as NF‐κB, MAPK, aryl hydrocarbon receptor (AhR), and peroxisome proliferators activate receptors‐γ (PPARγ), while also improving gut microbiota. These actions help increase TJ protein levels, reduce gut permeability, and lower inflammation. Preclinical and clinical studies suggest that polyphenols may help treat conditions like inflammatory bowel disease (IBD) by restoring gut health and immune balance. In the present review, we highlight how the Th17/Treg balance affects intestinal permeability, and recent findings regarding possible effects and the molecular mechanism of polyphenols in regulating the Th17/Treg balance, to provide new potential therapeutic strategies for damaged intestinal barrier treatment preclinically and clinically.

## Graphical abstract

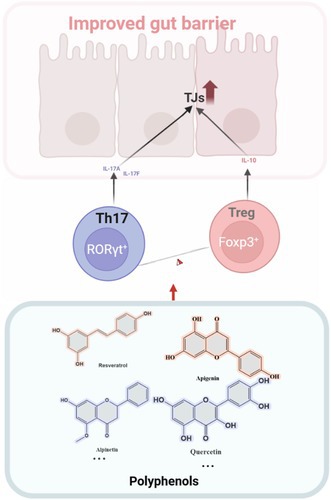

## 1 Introduction

Maintaining the integrity of the intestinal barrier is essential for overall health (Citi 2018). Disruption of TJ proteins increases intestinal permeability, allowing bacteria, endotoxins, and macromolecules to enter the bloodstream, which can lead to the development of autoimmune and metabolic diseases (Turner 2009). Despite its clinical significance, no drugs have been approved to specifically treat intestinal barrier dysfunction, and the underlying regulatory mechanisms remain unclear (Suzuki 2020). Emerging evidence suggests that intestinal Th17 and Treg cells play a critical role in the dynamic regulation of TJ assembly and disassembly (Blaschitz and Raffatellu 2010). This indicates that targeting the Th17/Treg imbalance could be an effective strategy to restore TJ function and enhance intestinal barrier integrity. Polyphenols, a diverse group of plant‐derived compounds, including flavonoids, phenolic acids, lignans, and tannins, have attracted increasing attention due to their wide‐ranging biological activities (Ahmad et al. 2022). Recent studies have demonstrated that many polyphenols can modulate Th17/Treg balance, promote TJ expression, and improve intestinal barrier function. In contrast, other natural compounds have shown limited potential in this regard, primarily due to the lack of fully elucidated mechanisms, low bioavailability, poor stability in vivo, and challenges associated with purification and standardization (Sorrenti et al. 2020). Furthermore, the relatively limited research attention and investment in these compounds have hindered systematic investigation and development. As a result, polyphenols have emerged as more promising candidates due to their established efficacy in regulating Th17/Treg homeostasis and enhancing TJ protein expression. Therefore, we highlight the interactions between Th17/Treg cells and TJs and the different mechanisms regulating Th17/Treg balance. Importantly, we also summarize the polyphenols that have the potential to improve intestinal barrier function by regulating Th17/Treg homeostasis and their molecular mechanisms.

## 2 Role of Th17/Treg Balance in Intestinal Barrier

### 2.1 Composition of the Intestinal Barrier

The intestinal barrier is a multilayered defense system comprising epithelial cells, TJs complexes, and the mucus layer, which collectively prevent pathogen infiltration and maintain intestinal homeostasis alongside systemic immune equilibrium (König et al. 2016; Untersmayr et al. 2022). Key biomarkers of intestinal barrier function include lipopolysaccharide (LPS), a direct indicator of bacterial translocation and endotoxemia in systemic circulation, and D‐lactate, a bacterial‐derived metabolite reflecting gut permeability alterations. While these markers are widely utilized, their interpretation requires context‐dependent validation and may be complemented by additional clinical or molecular assessments (Zhang, Li, et al. 2020). TJs on intestinal epithelial cells (IECs) comprise transmembrane proteins (e.g., Claudin and Occludin), peripheral scaffold proteins (such as ZO‐1), and regulatory proteins, all critical for preserving the barrier's structural and functional integrity. Consequently, circulating levels of TJ proteins are key indicators of intestinal barrier health (Ahmad et al. 2017). Compromise of the intestinal barrier reduces TJ protein expression, increases permeability, and allows toxins or pathogens to enter systemic circulation—a condition termed “leaky gut”. This disruption enables undigested antigens to translocate into the bloodstream (Kinashi and Hase 2021), triggering inflammation in gut and peripheral tissues and contributing to disorders such as IBD and irritable bowel syndrome (IBS). To defend against pathogens, IECs coordinate with lamina propria immune cells—such as monocyte‐derived macrophages, T lymphocytes, and innate lymphoid cells (ILCs)—to establish a robust defense mechanism (Johansson and Hansson 2016). The intestinal immune system sustains a delicate equilibrium between immune tolerance and inflammation through continuous interactions with microbial and dietary antigens (Doan et al. 2024). Disruption of this balance may result in immune dysregulation, heightening susceptibility to immune‐mediated inflammation and autoimmune diseases. Proinflammatory Th17 cells and regulatory Treg cells play pivotal roles in this dynamic (Zhao et al. 2022). Additionally, dysbiosis can activate immune cells, driving elevated production of proinflammatory cytokines (e.g., IL‐12, IL‐23, type I interferons) while suppressing anti‐inflammatory cytokines (e.g., TGF‐β, IL‐10) (Zhang, Chen, et al. 2020). This cytokine imbalance exacerbates intestinal barrier degradation and accelerates disease progression.

### 2.2 Th17 Cells, Treg Cells, and Intestinal Barrier

Th17 cells, a subset of CD4^+^ T cells, are defined by their expression of transcription factors RORγt/RORα and STAT3. These cells primarily secrete IL‐17A and IL‐17F, along with other cytokines such as IL‐21, IL‐22, IL‐26, IL‐8, and CCL20 (Cerboni et al. 2021). Th17 cells are a double‐edged sword that protects the integrity of the intestinal barrier in a non‐inflammatory manner. However, they usually act as attackers. As inflammatory inducers, Th17 cells could exacerbate the intestinal inflammatory response in the presence of pro‐inflammatory cytokines such as IL‐6, IL‐23, and IL‐1β, and become drivers of intestinal barrier disruption (Huber et al. 2012). Under physiological conditions, IL‐17A promotes the proliferation of IECs, enhancing barrier integrity. In addition to promoting epithelial repair, IL‐17A induces goblet cell differentiation, which increases mucus production and strengthens the epithelial barrier against microbial invasion (Leonardi et al. 2022). In a mouse model of DSS‐induced acute colitis, IL‐17A was found to promote epithelial barrier function by regulating the expression of the TJs protein via the adaptor protein Act1, thereby avoiding excessive increases in intestinal permeability. Interestingly, during the TNF‐α‐induced increase in Caco‐2 cell barrier permeability, the integrity of the intestinal epithelium was compromised without the presence of IL‐17A, and this effect could be reversed by the addition of IL‐17A. The above findings suggested that IL‐17A had a protective effect on the maintenance of intestinal epithelial integrity (Lee et al. 2015). In addition, IL‐17A and IL‐17F administration reversed the HIV‐1 gp140‐induced decrease in transepithelial electrical resistance (TEER) levels in Caco‐2 monolayers. In contrast, IL‐17A and IL‐17F, alone or in combination, could upregulate the expression of the TJs proteins Claudin‐1, Occludin, and ZO‐1. IL‐17A and IL‐17F also play roles in repairing intestinal epithelial injury through NF‐κB and MAPK‐dependent signaling pathways (Wang et al. 2019). Additionally, IL‐17A can rescue IFN‐γ‐induced disruption of TJs function by reactivating phosphorylated atypical protein kinase‐c ‐ζ (aPKC‐ζ), which is crucial in the early stages of TJs formation and for the maintenance of TJ function (Mizutani et al. 2021). However, under pathological conditions, when the immune balance is disrupted, a group of pathogens would invade the intestinal barrier and promote the production of pro‐inflammatory cytokines such as TGF‐β, IL‐6, IL‐23, and IL‐21 (Sundrud and Trivigno 2013). These cytokines induce T cells to differentiate into Th17 cells with the help of antigen‐presenting cells such as dendritic cells. At this point, IL‐17A and IL‐17F secreted by Th17 cells exhibit pathogenicity and disrupt the intestinal barrier. In an in vitro model of the intestinal epithelium using Caco‐2 cell monolayers, Rahman et al. (2018) demonstrated that IL‐17A increased intestinal barrier permeability by reducing the expression of the TJs proteins ZO‐1, Claudin‐5, and Occludin, which may be associated with the heterogeneous assembly of the surrounding F‐actin cytoskeleton. Another in vivo study has shown that inhibition of IL‐17F could protect mice from DSS and T cell‐induced colitis. Similarly, Tang et al. (2018) showed that the antimicrobial peptides (β‐defensin 1 and β‐defensin 4) were significantly enhanced in the colon of *Il‐17f* ^−/−^ mice compared to wild‐type mice. Furthermore, to determine the relationship between intestinal permeability and the distribution of lymphocytes in the intestine and secondary lymphoid organs, Alba Miranda‐Ribera et al. overexpressed zonulin, an important regulator of intestinal epithelial TJs proteins, to establish a transgenic mouse model with impaired primary intestinal barrier function (Ztm). They found that the major immune cell subpopulations were not significantly altered in Ztm mice compared to wild‐type mice. However, the increased proportion of IL‐17–producing innate and innate‐like cells in the intestine and spleen of Ztm mice suggested that the immune response threshold in Ztm mice may be altered. The threshold of immune reactivity in Ztm mice might be altered to make them more susceptible to lose tolerance to non–self‐antigens, which in turn leads to more severe colitis (Miranda‐Ribera et al. 2019).

Treg cells, another CD4^+^ subset enriched in gut‐associated lymphoid tissue (GALT), maintain barrier integrity via anti‐inflammatory cytokines like IL‐10 and TGF‐β (Chaudhry et al. 2011; Geem et al. 2016; Rubtsov et al. 2008; Song et al. 2020). Studies have shown that Treg cells produce anti‐inflammatory cytokines such as IL‐10 and transforming growth factor‐β (TGF‐β) to alleviate increased epithelial cell permeability caused by *Escherichia coli* infection (Howe et al. 2005). IL‐10 signals through the IL‐10 receptor (IL‐10R), which consists of two α subunits (IL‐10R1 and IL‐10RA) and two β subunits (IL‐10R2 and IL‐10RB) (Kotenko et al. 1997). The α subunits are central to the formation of the intestinal barrier and have an important role in maintaining and restoring the intestinal barrier. Reduction of IL‐10RA leads to a loss of barrier‐forming function in the intestinal epithelium (Bhutiani et al. 2018; Saraiva et al. 2020). Kominsky et al. (2014) used a lentiviral transduction system to reduce IL‐10R1 expression in human T84 human epithelial cell (IEC) and found that its barrier‐forming function was significantly reduced. Similar results were also observed in another research; Zheng et al. (2017) found that IL‐10RA inhibited the expression of Claudin‐2, thereby increasing intestinal permeability. In summary, IL‐10RA plays a crucial role in the formation and maintenance of intestinal barrier integrity, and the role of IL‐10 in helping to establish, preserve, and restore the integrity of the intestinal barrier is virtually undisputed (Table 2).

**FIGURE 1:**
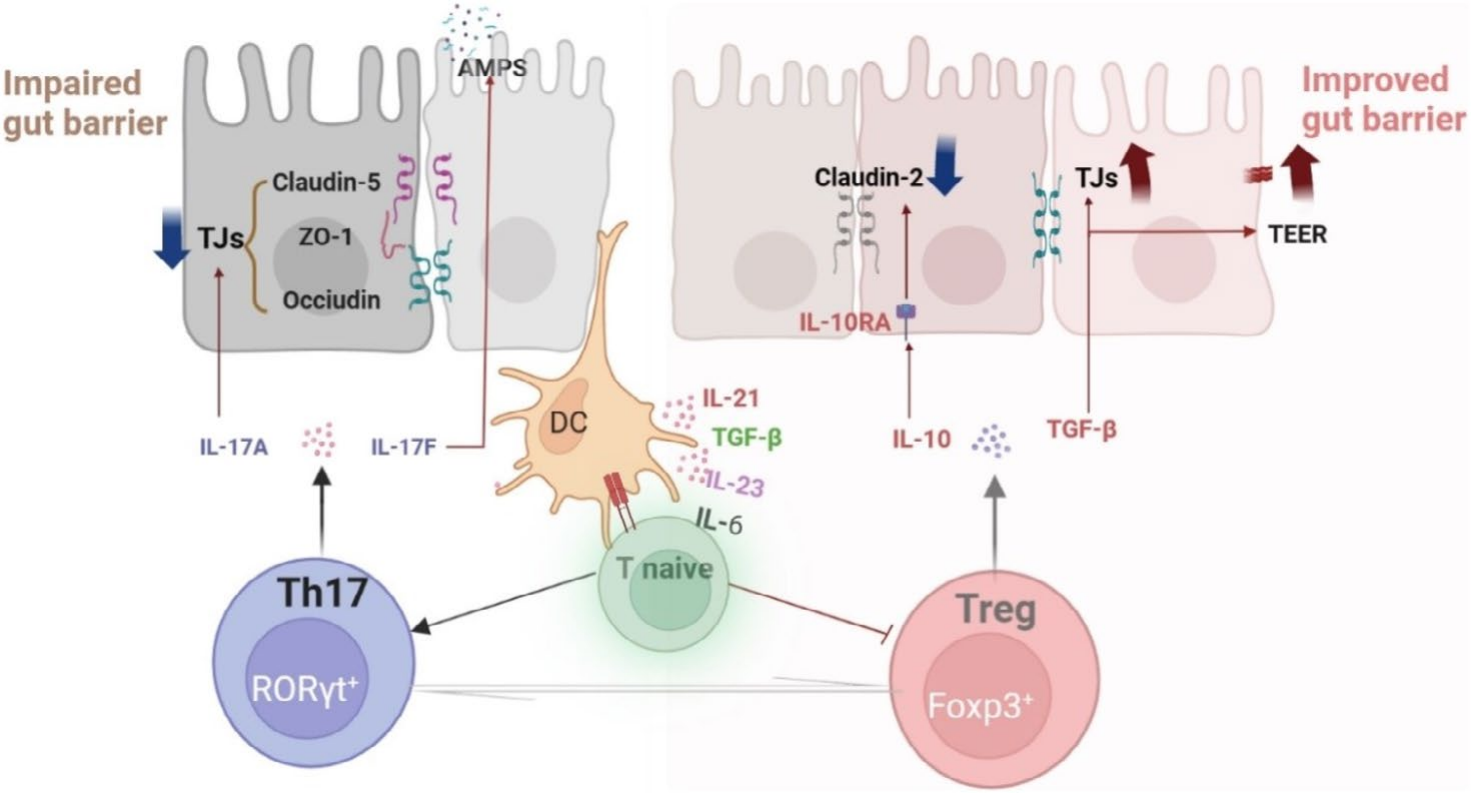
The interaction mechanism between Th17/Treg and the intestinal barrier. Th17 cells impair gut barrier function by secreting IL‐17A and IL‐17F, which inhibit TJs such as Claudin‐5, ZO‐1, and Occludin. DCs promote Th17 differentiation through IL‐6 and IL‐23, enhancing the inflammatory response. Treg cells improve gut barrier repair by secreting IL‐10 and TGF‐β, inhibiting Claudin‐2 expression, strengthening tight junctions, and enhancing gut barrier function to maintain intestinal integrity and function.

In short, these findings underscore the Th17/Treg axis as a central regulator of intestinal barrier integrity, orchestrating dynamic TJs assembly, epithelial repair, and immune‐microenvironment crosstalk (Figure 1). Disruption of this balance initiates a vicious cycle of barrier breakdown and dysbiosis‐driven inflammation, providing a mechanistic rationale for immunomodulatory therapies targeting Th17/Treg polarization in barrier‐related pathologies.

## 3 Strategies Targeting Th17/Treg Balance

### 3.1 Signaling Pathways

#### 3.1.1 Peroxisome Proliferators Activate Receptors‐γ

**FIGURE 2:**
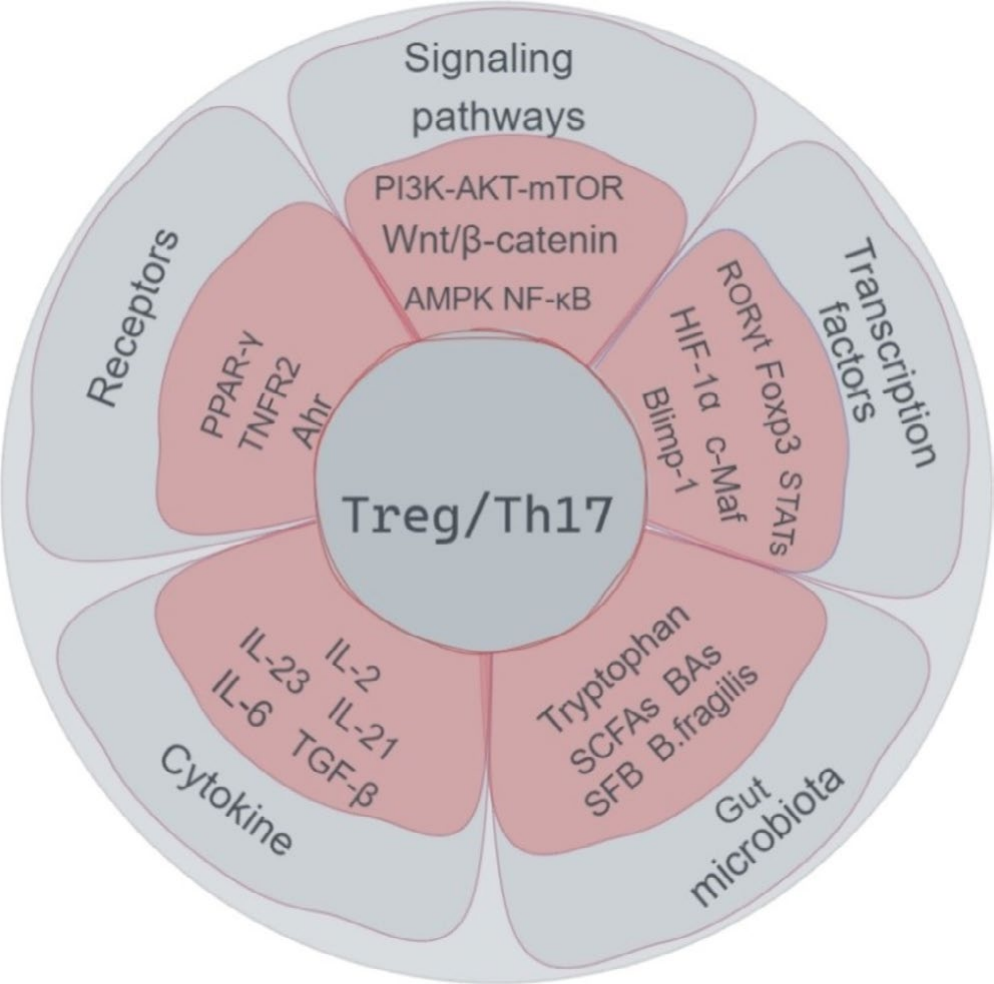
Regulatory strategies targeting Th17/Treg balance. The Th17/Treg balance is regulated by multiple molecular mechanisms, including signaling pathways, receptors, transcription factors, cytokines, and gut microbiota. Key signaling pathways include PI3K‐AKT–mTOR, Wnt/β‐catenin, AMPK, and NF‐κB. Important receptors include PPAR‐γ, TNFR2, and AhR. Key transcription factors are RORγ, FoxP3, STATs, HIF‐α, c‐Maf, and Blimp‐1. Important cytokines include IL‐2, IL‐6, IL‐21, IL‐23, and TGF‐β. These molecules act in coordination to regulate the differentiation and function of Treg and Th17 cells, maintaining immune balance.

Peroxisome proliferators activate receptors‐γ (PPAR‐γ) is a key regulator of Th17 and Treg cell differentiation (Xu et al. 2017). It not only promotes Treg cell differentiation but also inhibits Th17 cell development. It has been shown that PPAR‐γ selectively inhibits Th17 differentiation in a T‐cell intrinsic manner (Klotz et al. 2009). PPAR‐γ has been shown to inhibit *Il‐17a* expression by recruiting the nuclear receptor corepressor (NcoR) and the silencing mediators of retinoid and thyroid hormone receptors (SMRT) to the RORγt promoter and blocking transcription (Park and Pan 2015). Yang, Zheng, et al. (2022) found that Bergenin, an agonist of PPAR‐γ, reduced the percentage of Th17 cells and the expression of RORγt, IL‐17A/F, IL‐21, and IL‐22 in naïve T cells under Th17‐polarizing conditions. In contrast, the PPAR‐γ antagonist GW9662 and siPPAR‐γ abrogated the inhibitory effect of Bergenin on Th17 differentiation. In addition, PPAR‐γ promotes Treg cell proliferation, mediates the conversion of Th17 cells into Treg cells, and induces Treg cell differentiation by upregulating CD36/CPT1‐mediated fatty acid oxidation (Miao et al. 2022). Similarly, Cipolletta et al. (2012) observed reduced Treg cell expression in PPAR‐γ‐deficient mice. Therefore, it is possible to regulate Th17 and Treg cells by regulating PPAR‐γ (Figure 2).

#### 3.1.2 Aryl Hydrocarbon Receptor

Aryl hydrocarbon receptor (AhR) is a ligand‐activated transcription factor that induces Treg cell differentiation and inhibits Th17 cell differentiation in a ligand‐specific manner. A high‐affinity ligand of AhR, 2,3,7,8‐tetrachlorodibenzo‐p‐dioxin (TCDD), was reported to upregulate Treg cell expression and inhibit Th17 cell differentiation by mediating Foxp3 promoter demethylation and IL‐17 promoter methylation, resulting in elevated Foxp3 expression and decreased IL‐17 expression (Singh et al. 2011). Additionally, many natural substances can also act directly as ligands of AhR to regulate the Th17/Treg balance, such as Baicalein, a flavonoid primarily derived from the roots of *Scutellaria baicalensis*, a traditional Chinese medicinal herb, which can promote AhR activation and induce its translocation to the nucleus, and increase the expression level of Cytochrome P450 1A1 Enzyme (CYP1A1), a downstream protein of AhR, to induce Treg cell differentiation and inhibit Th17 cell differentiation (Liu et al. 2020). As such, AhR may be a target for regulating Th17 and Treg homeostasis, based on its ligand‐regulatory properties.

#### 3.1.3 Tumor Necrosis Factor Receptor 2

Tumor necrosis factor receptor 2 (TNFR2), a member of the TNF superfamily, is expressed on the surface of Th17 and Treg cells and mediates the pro‐ and anti‐inflammatory activity of T cells (Tam et al. 2019). TNFR2 is a reliable phenotypic and functional surface marker of Treg cells. Through interaction with TNF, it plays a decisive role in Treg cell activation, expansion, and phenotypic stability. TNFR2 agonists have been repeatedly shown to enhance Treg function, while TNFR2 antagonists can inhibit Treg cell proliferation. When the TNF/TNFR2 signaling pathway is activated, it can enhance IL‐2 production by Treg cells and effector T cells, and IL‐2 can further inhibit Th17 cell differentiation. When the TNF/TNFR2 signaling pathway is blocked or impaired, IL‐2 production is reduced, STAT3 activity and RORγt levels are increased to enhance Th17 cell differentiation, while STAT5 activity is reduced to decrease Treg cell differentiation (Miller et al. 2015). In addition, TNFR2 interacts with TNFα to enhance the activation of Akt in the PI3K/Akt pathway, which further increases STAT5 phosphorylation and thus enhances the differentiation of Treg cells, while decreasing the differentiation of Th17 cells (Yang et al. 2018). Therefore, controlling Treg functional signaling by targeting TNF‐TNFR2 can be used to regulate Th17/Treg homeostasis.

#### 3.1.4 PI3K‐AKT–mTOR Signaling Pathway

The impact and mechanism of action of PI3K on Th17/Treg homeostasis may be multifaceted. Over the past decade, many studies have demonstrated the important role of the PI3K/AKT pathway in regulatory T cell development, function, and stability. One of the most intensively explored is the promotion of mTOR activity, which is located downstream of the PI3K/Akt pathway and regulates Th17/Treg homeostasis by integrating transcriptional and metabolic programs. The mTOR complex includes two distinct multimolecular signaling forms, mTORC1 and mTORC2. mTORC1 is the primary signal required for Th17 cell differentiation (Sasaki et al. 2016). Previous studies have shown that the PI3K‐Akt‐mTORC1‐S6K1/2 axis positively regulated Th17 cell differentiation by decreasing the transcription factor growth factor independence 1 (Gfi1) expression and promoting nuclear translocation of RORγt (Kurebayashi et al. 2021). In addition, mTORC1 may inhibit Treg cell differentiation by phosphorylating STAT3 to drive IL‐17 expression while inhibiting smad3 phosphorylation or H3K4 methylation near the Foxp3 transcription start site (TSS) and within the 5′ untranslated region (UTR) (Sauer et al. 2008). Sb et al. reported that the mTORC1 inhibitor, rapamycin, could inhibit STAT3 phosphorylation and IL‐17 expression, induce Foxp3 expression, and drive functional Treg cell expansion in vitro. Furthermore, in the metabolic pathway, mTORC1 triggers glycolysis in Th17 cells through activation of the glycolysis‐promoting transcription factor HIF‐1α. In contrast, in contrast to Th17 cell differentiation, Treg cells are less dependent on glycolytic activity and glycolytic enzymes. Therefore, reducing glycolysis in T cells by inhibiting the PI3K‐Akt‐mTORC1 signaling pathway may be an effective way to inhibit the differentiation of Th17 cells while promoting Treg differentiation.

#### 3.1.5 AMPK Signaling Pathway

AMPK‐activated protein kinase is a heterotrimeric kinase complex consisting of a catalytic α‐subunit, a regulatory β‐subunit, and an AMP‐binding γ‐subunit (Yan et al. 2018). Among them, the γ subunit of AMPK can detect changes in the AMP/ATP ratio, and if an increase in the ratio is detected (energy deficit), AMPK will be activated, and the body will downregulate energy‐consuming metabolism such as glycolysis, while energy‐producing catabolism will be upregulated, promoting mitochondrial oxidative phosphorylation and fatty acid oxidation. Th17 cell differentiation is more dependent on glycolysis, while Treg cells are more dependent on fatty acid oxidation. Thus, this ability of AMPK to regulate metabolism could affect the balance of Th17/Treg. AMPK is highly expressed in Treg cells, and activated AMPK drives differentiation of naive T cells to Treg cells by enhancing fatty acid oxidation in vitro and in vivo. Recent studies show that the direct activator of AMPK, AICAR (5‐aminoimidazole‐4‐carboxamide ribonucleotide), improved Th17/Treg homeostasis by activating fatty acid oxidation. In contrast, the indirect activator of AMPK, metformin, was reported to inhibit T cell proliferation and suppress Th17 cell differentiation while promoting Treg cell development in a dose‐dependent manner in vitro (Bai et al. 2010; Gualdoni et al. 2016). On the other hand, AMPK activation also inhibits mTOR signaling, leading to the production of Treg cells. In contrast, defective AMPK function usually results in increased mTOR activity and upregulation of glycolysis, which increases effector Th17 production (Shen et al. 2020). In summary, AMPK is a key signaling pathway that determines the direction of T cell differentiation, and targeting AMPK has the potential to regulate the Th17/Treg balance.

#### 3.1.6 Notch Signaling Pathway

The Notch signaling pathway is an evolutionarily conserved intercellular signaling cascade that is extensively involved in T cell development and differentiation (Siebel and Lendahl 2017). It contains four Notch receptors (Notch1‐4), which are activated by binding to five transmembrane binding ligands (sDLL1, 3, 4 and Jagged1, 2) (Grazioli et al. 2017). Activation of the Notch signaling pathway usually causes a Th17/Treg imbalance. Studies have shown that increased Jagged1 and Delta4 can effectively elevate the Th17/Treg ratio. In addition, the Notch signaling pathway can also directly and positively regulate the differentiation of Th17 cells. For example, Notch1 was found to promote Th17 differentiation by directly binding to the promoters of *Il‐17* and *Rorɣt* (Coutaz et al. 2016). Consistent with this study, the siRNA of Notch1 also was able to reduce the production of IL‐17. In addition to Notch1, sDLL4 was reported to enhance IL‐17 production in the presence of TGF‐β and IL‐6. Further studies revealed that this effect was due to the typical Notch signaling molecule RBPJ, which directly interacted with RORγt and the IL‐17 promoter (Vijayaraghavan and Osborne 2018). On the other hand, the Notch signaling pathway is involved in inhibiting the differentiation and development of Treg cells not only by downregulating Foxp3 expression but also by regulating STAT5 phosphorylation. Thus, blocking Notch1 signaling normally leads to an increase in Treg cells. In a mouse model, Treg cells in the spleen were greatly increased after the Notch1/Jagged1 signaling pathway was inhibited by the inhibitor GSI, suggesting that Notch1/Jagged1 inhibited Treg cell differentiation (Jiao et al. 2019). Therefore, the Th17/Treg balance can be regulated by inhibiting the Notch pathway.

#### 3.1.7 NF‐κB Signaling Pathway

The NF‐κB signaling pathway is involved in T cell activation and is essential for Treg and Th17 cell development. The NF‐κB family consists of five subunits, RelA (p65), RelB, c‐Rel, NF‐κB1 (p105), and NF‐κB2 (p100). It was shown that Th17 differentiation was controlled by the cRel‐RORγ‐RORγt axis, in which c‐Rel and RelA/p65 induced Th17 differentiation by binding to and activating two different Rorg promoters. In contrast, in c‐Rel or RelA‐deficient T cells, Th17 differentiation was impaired, while the gene expressions of RORγt and RORγ were significantly reduced (Ruan et al. 2011). Thus, targeting Rel/NF‐κB is effective in controlling Th17 cell‐mediated disease. c‐Rel is also important for the development of thymic Treg cells (Ziegler et al. 2021). It could bind to CNS3, a non‐coding sequence of FoxP3, and promote thymic and peripheral Treg cell differentiation. In contrast, knockdown of c‐Rel has been evidenced to impair Treg development (Zheng et al. 2010). A significant reduction in the number of Treg cells or impaired differentiation of Treg cells was observed in c‐Rel‐deficient mice or in c‐Rel‐deficient T cells.

#### 3.1.8 Wnt/β‐Catenin Pathway

The Wnt/β‐catenin signaling pathway is an evolutionarily conserved pathway that regulates the differentiation and function of CD4^+^T cells. β‐catenin is the core effector of this pathway, which is related to many Foxp3‐binding genes, so the activated Wnt signaling pathway can directly regulate Foxp3 activity and thus inhibit Treg cell function (Quandt et al. 2021). BIO is a widely used inhibitor of the Wnt signaling pathway; it significantly reduced the levels of CD25, which were regulated by Foxp3 in human Treg cells (van Loosdregt et al. 2013). In addition, the Wnt/β‐catenin signaling pathway can also exacerbate disease progression by enhancing RORγt activation and Th17 differentiation. In a lipopolysaccharide‐induced acute lung injury model, agonists of the Wnt/β‐catenin pathway, LiCl, enhanced Th17 cell proliferation and IL‐23 expression. However, Dkk‐1, an inhibitor of this pathway, greatly reduced Th17 cell activation, suggesting that activation of the Wnt/β‐catenin pathway enhanced the Th17 response. Further studies showed that activation of the Wnt/β‐catenin pathway induced RORγt expression via the histone acetyltransferase p300, which could be reduced by the inhibitor of p300, C646. The above findings suggest that the Wnt/β‐catenin pathway has an important role in inducing inflammation by suppressing Treg cell function and promoting Th17 differentiation (Quandt et al. 2021).

### 3.2 Gut Microbiota and Its Metabolism

Over the past decades, many studies in germ‐free (GF) mice have shown that the microbiota played an integral role in shaping the host intestinal immune system. Meanwhile, these studies also have shown that the homeostasis between Th17 and Treg cells in the gut was dependent on the composition and species of gut microbes. Microbial metabolites, small molecules produced as intermediates or end products of microbial metabolism, are considered one of the main modes of interaction between the gut microbiota and the host, and their role in regulating CD4^+^ T cells has been intensively studied.

#### 3.2.1 Gut Microbiota

Segmentous filamentous bacteria (SFB) are anaerobic gram‐positive bacilli that are essential for the proliferation and activation of Th17 cells. Studies have shown that SFB colonization in the intestine could lead to an increase in the number of Th17 cells and a decrease in the number of Treg cells, possibly because SFB was able to promote serum amyloid proteins (SAA) production in the ileum, thereby inducing Th17 cell differentiation (Atarashi et al. 2015). Another study also found that various cytokines, including IL‐1β, IL‐6, and IL‐23, which were secreted by CD11c^+^ cells after SAA stimulation, could promote Th17 cell differentiation together with SAA (Bang et al. 2021). In contrast, another commensal bacterium, *Bacteroides fragilis* ( *B. fragilis* ), could inhibit IL‐17 production and enhance intestinal Treg cell activity by producing polysaccharide A (PSA) with anti‐inflammatory effects (Jin et al. 2012). PSA is an immunomodulatory molecule present in the pod membrane of *B. fragilis* , which mediates the conversion of CD4^+^ T cells into Treg cells via toll‐like receptor 2 (TLR2) (Round et al. 2011). In addition, PSA is recognized by dendritic cells (DCs) in the intestine and then causes IL‐10 production by DC cells, thus promoting Treg production (Chu et al. 2016). In addition, many microorganisms such as *Clostridium perfringens* , *Staphylococcus saprophyticus* , *Bacillus mimicus*, and *Clostridium histolyticum* have the potential to induce pTreg cell production (Ohnmacht 2015; Xu et al. 2018). *Clostridium perfringens* cluster IV and XIVa have been proven to induce the accumulation of Treg cells in colonic LP, and the mechanism of this induction may be closely related to its metabolite, short‐chain fatty acids (SCFAs) (Livanos et al. 2018).

#### 3.2.2 Short‐Chain Fatty Acids

Short‐chain fatty acids (SCFAs) are fatty acids containing less than 6 carbon atoms and are mainly produced in the colon by gut microbiota after fermentation of carbohydrates, including acetic acid, propionic acid, and butyric acid, etc. Many in vitro studies have shown that SCFAs were able to initiate Treg differentiation while suppressing the proliferation of pathogenic Th17 cells. For example, Park et al. (2015) found that *Rag1* ^−/−^ mice (colitis model) injected with acetate‐pretreated Th17 cells had less severe symptoms than those injected with untreated Th17 cells, possibly due to higher levels of IL‐10 expression in Th17 cells after acetate intervention. Propionic acid and butyric acid were also shown to promote IL‐10 production and inhibit IL‐17 secretion, with butyric acid having the lowest concentration required to exert its effect.

The effect of SCFAs on regulating Th17/Treg balance may be related to their ability to inhibit histone deacetylase (HDAC) and GPR43 signaling (Furusawa et al. 2013). A recent study showed that propionic acid could upregulate GPR43 levels while downregulating HDAC6 expression to inhibit CD4^+^T cell differentiation into Th17 cells and promote Treg cell differentiation. Sun et al. also observed that butyric acid inhibited HDAC and GPR3 signaling in a dose‐dependent manner, directly reducing the proliferation of Th17 cells and IL‐17 production in the lamina propria of the intestine. Similarly, in CD4^+^T cells, valeric acid also inhibited HDAC activity and reduced IL‐17A expression (Sun et al. 2017). And interestingly, although valeric acid did not promote Treg cell proliferation, it significantly increased IL‐10 levels in Treg cells, which may be related to the enhanced glucose oxidation. However, a recent study showed that both Treg and Th17 cells were significantly increased in the colon of IBD model mice after oral administration of butyric acid or a mixture of SCFAs, which could not effectively alleviate colonic inflammation. This contradictory result may be related to the concentration of intervention and route of administration of SCFAs. These studies suggest that SCFAs may inhibit Th17 cell differentiation and promote Treg cell proliferation or IL‐10 expression through epigenetic and metabolic regulation, but the administration ways and intervention concentration of SCFAs in mice and even humans still deserve further investigation.

#### 3.2.3 Bile Acids

Another class of bacterial metabolites, bile acids (BAs), has also been shown to be essential for the maintenance of colonic RORγt^+^ Treg cells (Campbell et al. 2020). Many BAs have affinity for intestinal farnesol X receptor (FXR) and G protein‐coupled bile acid receptor (TGR5), and activate Treg cells by promoting the production of anti‐inflammatory cytokines such as IL‐10, while reducing the secretion of pro‐inflammatory cytokines IL‐6, IL‐1β, TNF‐α and IL‐17 and thus inhibiting Th17 activation (Ding et al. 2015). BAR501 is a bile acid derivative that selectively activates GPBAR1, a cell membrane receptor for secondary bile acids, which is essential for maintaining intestinal immune homeostasis. It has been reported that BAR501 alleviated TNBS‐induced colitis by acting on GPBAR1 to increase the number of colonic Treg cells and IL‐10 expression levels, which was not observed in IL‐10 knockout mice. In addition, Saiyu Hang et al. found that two other derivatives of lithophanic acid, 3‐oxoLCA and isoalloLCA, had effects on regulating Th17/Treg balance (Hang et al. 2019). They observed that 3‐oxoLCA inhibited Th17 cell differentiation by directly binding to RORγt, while isoalloLCA enhanced Treg differentiation by producing mitochondrial reactive oxygen species (mitoROS) leading to increased FoxP3 expression. These studies suggested that BAs can control the host intestinal immune response by regulating the balance of Th17 and Treg.

#### 3.2.4 Tryptophan

Tryptophan, an essential aromatic amino acid, is one of the metabolites that are important in the exchange between the intestinal flora and the host. Tryptophan metabolites such as kynurenine, indole, and indole derivatives are regulated directly or indirectly by the gut microbiota and play a critical role in regulating intestinal mucosal immunity and maintaining homeostasis in the intestine. Tryptophan metabolites have been reported to regulate the balance between Treg and Th17 by binding to and activating the AhR, thereby modulating the local immune response (Zelante et al. 2013). AhR ligands consist of endogenous metabolites (including kynurenine, kynurenic acid, xanthurenic acid, and cinnamic acid) and bacterial metabolites (including indole, indolepropionic acid, indoleacetic acid, fecal odorant, and tryptamine). After activation of AhR, IL‐22 will be released and then induce IL‐10R to selectively enhance the production of Treg cells in vitro, thereby regulating intestinal homeostasis (Mezrich et al. 2010). Critically, AhR signaling exhibits microenvironment‐dependent duality: under inflammation, it promotes DC‐mediated immune tolerance, whereas, in homeostasis, it reinforces epithelial barrier integrity. This context‐specificity underscores the need for precise timing and pathology‐stage evaluation when developing AhR‐targeted therapies.

## 4 Polyphenols Targeting Th17/Treg Balance in Impaired Intestinal Barrier

**FIGURE 3:**
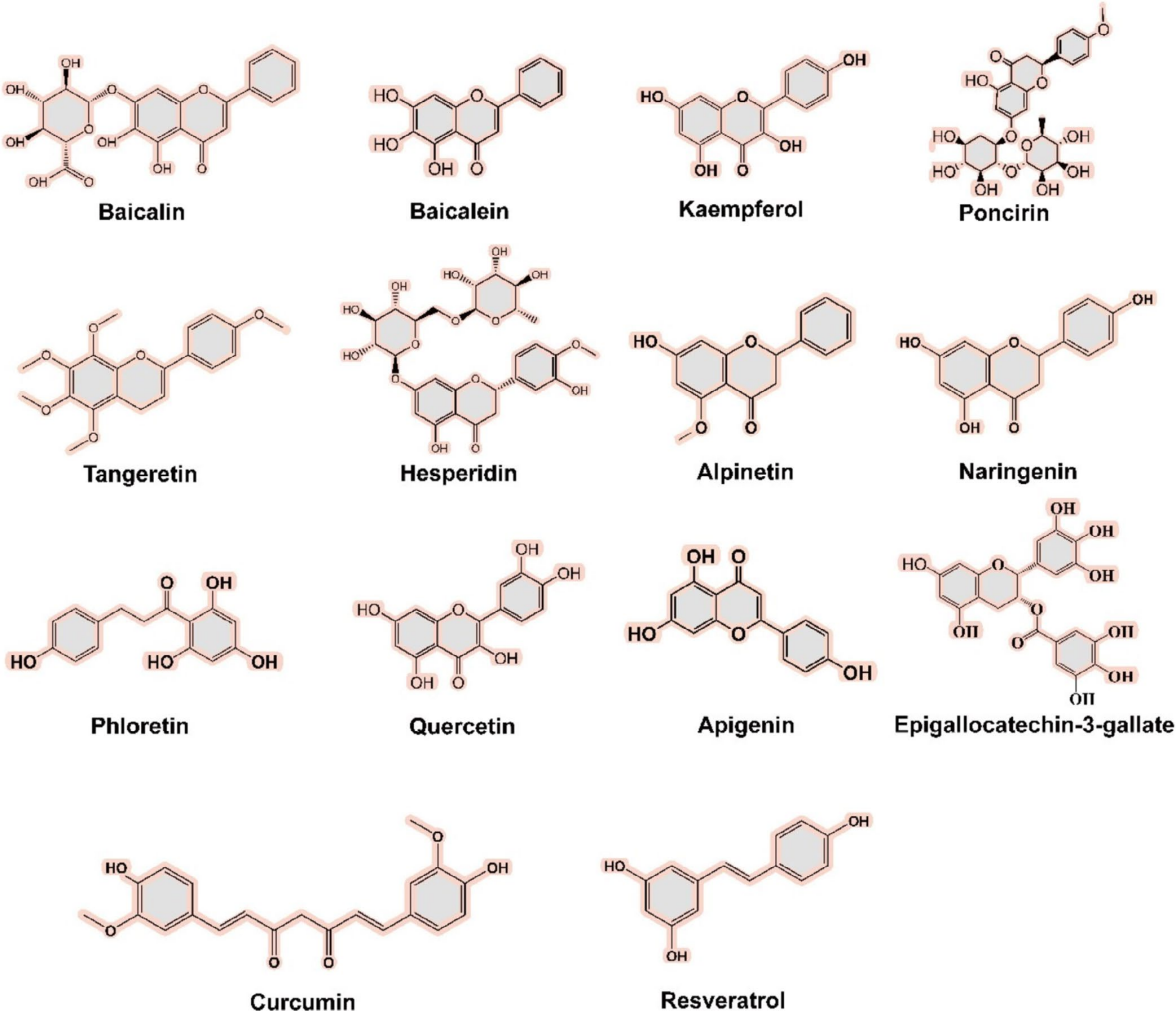
Structural formula of polyphenols for improving the intestinal barrier via impacting the Treg/Th17 balance.

**FIGURE 4:**
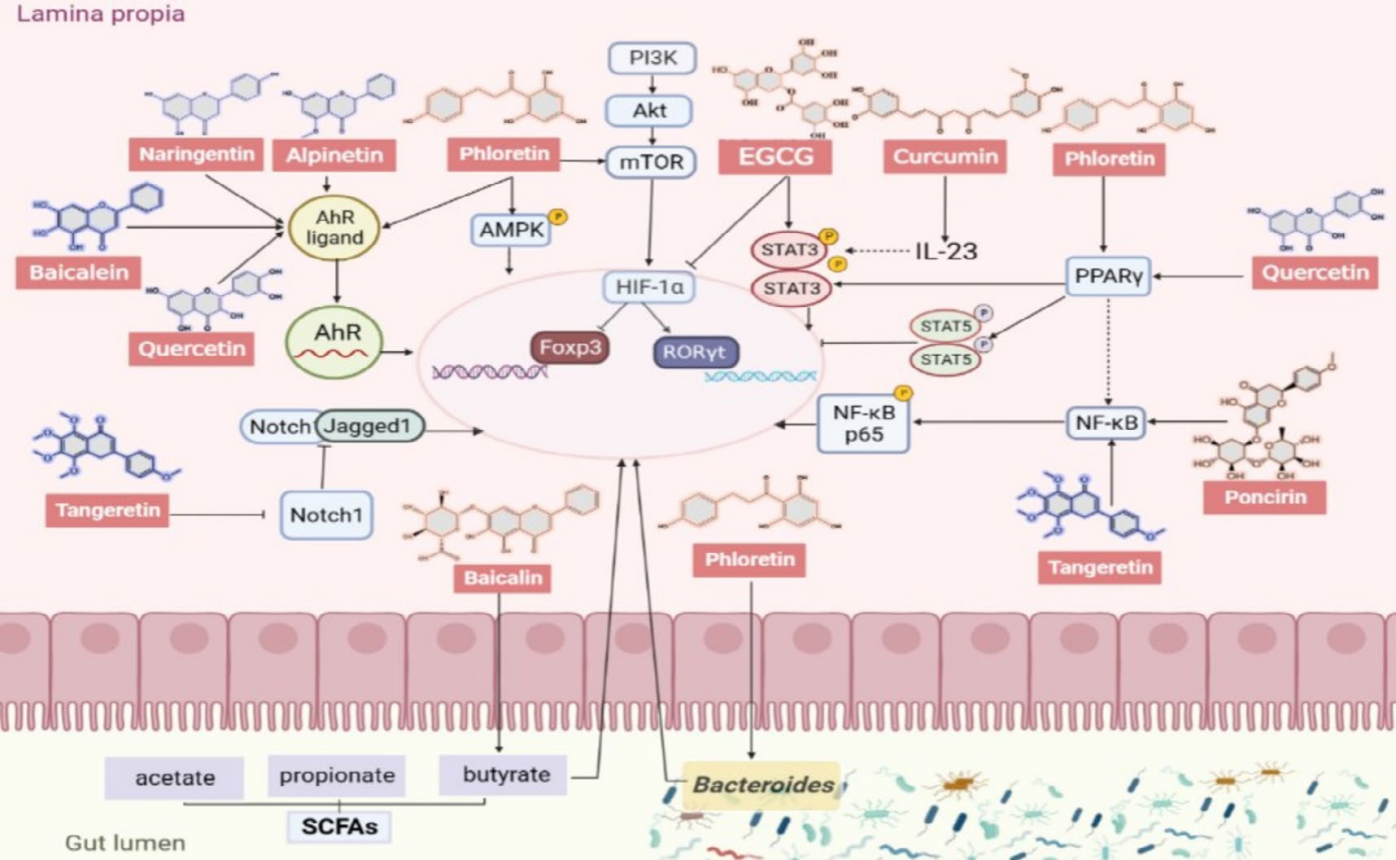
The mechanism of polyphenols improved the intestinal barrier by regulating Th17/Treg balance.

Polyphenolic compounds are widely found in various plants, fruits, and vegetables, including phenolic acids, flavonoids, astragals, and lignans. They can be mainly divided into 6 subgroups, namely flavonols, flavones, flavanones, isoflavones, anthocyanins, and flavanols. Despite their low systemic bioavailability due to limited intestinal absorption and extensive phase II metabolism (e.g., glucuronidation and sulfation), polyphenols exert significant biological effects through two pathways: (1) direct interaction with gut microbiota, where microbial enzymes (e.g., β‐glucosidases, esterases) hydrolyze glycosides and esterified forms into bioactive aglycones (Deng et al. 2025); (2) generation of microbial metabolites (e.g., phenyl‐γ‐valerolactones, phenolic acids) that enter systemic circulation via the portal vein or modulate local immune responses in the gut (Di Lorenzo et al. 2021). A recent meta‐analysis of the anti‐inflammatory mechanisms of plant‐derived natural compounds in the treatment of acute lung injury (ALI) further revealed the intestinal immunomodulatory potential of polyphenols. The results showed that dietary supplementation rich in polyphenols could significantly enhance the integrity of intestinal barrier function and reduce the level of pro‐inflammatory factors, potentially linked to their microbial‐driven metabolic transformation in the colon (Chen et al. 2025). In the past several decades, an increasing number of publications have reported that many polyphenols are able to modulate the Th17/Treg balance in the intestine, including Baicalin, Kaempferol, *Lycium barbarum* among others (Pavlova 2024) (Table 1). In this section, we review some important polyphenolic compounds targeting the Th17/Treg balance and their potential mechanisms (Figures 3 and 4).

**TABLE 1:** Polyphenols used to improve the intestinal barrier by regulating Th17/Treg.

Compounds	Model	Effective dose	IL‐10	IL‐17	Treg	Th17	Intestinal barrier	Pathways	References
Baicalin	TNBS‐induced colitis in SD rats	25, 50, 100 mg/kg/day	↑	**↓**	↑	**↓**	ZO‐1, Occludin and MUC2↑	ROR γ t↓ Foxp3↑ SCFA	Zhu et al. (2020)
Baicalein	DSS‐induced colitis in male C57BL/6 mice	10, 20, 40 mg/kg/day	—	—	—	—	ZO‐1, Occludin↑	AHR	Li et al. (2022)
	DSS‐induced colitis in male C57BL/6 mice	10, 20, 40 mg/kg/day	↑	**↓**	↑	**↓**	—	—	Liu et al. (2020)
Kaempferol	DSS‐induced colitis in female C57BL/6 mice	50 mg/kg/day	↑	—	—	—	ZO‐1, Occludin and Claudin‐1↑ Intestinal permeability↑	—	Qu et al. (2021)
Poncirin	TNBS‐induced colitis in male C57BL/6 mice	10, 20 mg/kg/day	↑	**↓**	↑	**↓**	ZO‐1, Occludin and Claudin‐1↑	RORγ t↓ Foxp3↑	Kang and Kim (2016)
Hesperidin	DSS‐induced colitis in male C57BL/6 mice	10, 20, 40 mg/kg/day	↑	—	↑	—	ZO‐1 and Occludin↑ Intestinal permeability↑	—	Guo et al. (2019)
Tangeretin	TNBS‐induced colitis in male C57BL/6 mice	10, 40 mg/kg/day	↑	**↓**	↑	**↓**	ZO‐1, Occludin and Claudin‐1↑	ROR γ t↓ Foxp3↑ NF‐κBp65	Eun et al. (2017)
	DSS‐induced colitis in male C57BL/6 mice	0.08% w/w	↑	—	—	—	ZO‐1 and Claudin‐1↑	ROR γ t↓ Foxp3↑	Chen et al. (2021)
	CD3/CD28‐induced splenocytes	4, 10, 18 μM	—	—	↑	—	—	Foxp3↑ Notch1↓	Xu et al. (2019)
Alpinetin	DSS‐induced colitis in male C57BL/6 mice	25, 50, 100 mg/kg/day	—	—	—	—	ZO‐1, Occludin and Claudin‐1↑ Claudin‐2**↓**	——	Tan and Zheng (2018)
	DSS‐induced colitis in female C57BL/6 mice	30 mg/kg/day	—	—	—	—	Intestinal permeability↑	Ahr↑	Miao et al. (2019)
	DSS‐induced colitis in female C57BL/6 mice	7.5, 15, 30 mg/kg/d	↑	**↓**	↑	**↓**	—	Ahr↑ Foxp3↑	Lv et al. (2018)
	Mouse naïve CD4^+^ T cells‐based Th17 and Treg differentiation in vitro	10, 30 μM	—	—	↑	**↓**	—	Ahr↑ Foxp3↑	
Naringenin	Caco‐2	100 μM	—	—	—	—	ZO‐1, ZO‐2, Occludin, Claudins and junctional adhesion↑ TEER↑	—	Noda et al. (2012)
	DSS‐induced colitis in female BALB/c mice	50 mg/kg/d	—	—	↑	—	—	Ahr↑ Foxp3↑	H. K. Wang et al. (2012)
	Mouse naïve CD4^+^ T cells‐based Treg differentiation in vitro	6.25, 12.5, 25, 50 μM	↑	—	↑	—	—	Ahr↑ Foxp3↑	Wang et al. (2012)
Phloretin	DSS‐induced colitis in male BALB/c mice	60 mg/kg/d	↑	—	↑	**↓**	Muc2, ZO‐1and Claudin‐1↑	Bacteroidete RORγt↓ Foxp3↑	Wu et al. (2019)
	DSS‐induced colitis in male C57BL/6 mice	25, 50, 100 mg/kg/d	—	—	—	—	serum LPS content↑	NF‐*Κ*B ↑ PPARγ↑	Zhang et al. (2019)
	Human peripheral blood CD4^+^ T cells	25, 50, 100 μM	—	—	↑	**↓**	—	p‐AMPK↑ PPARγ ↑ P‐STAT3↑ P‐STAT5↓	A. Jiao et al. (2020)
Quercetin	Acute necrotizing pancreatitis in male SD rat	50 mg/kg/d	—	—	—	—	ZO‐1, Occludin and Claudin‐1↑ DAO d‐lactate and endotoxin**↓**	——	Zheng et al. (2018)
	CIA model in male DBA/1 J mice	150 mg/kg/d	—	—	—	—	—	PPARγ↑ STAT3↑	Yang, Shi, et al. (2022b)
	DSS‐induced colitis in female C57BL/6 mice	50 mg/kg/d	—	—	—	—	—	—	Riemschneider et al. (2021)
Apigenin	HFD‐induced obese in male C57BL/6 mice	50 mg/kg/d	—	—	↑	—	Claudin‐1↑ Intestinal permeability↑	—	Qiao et al. (2021)
EGCG	CTX‐treated in Male ICR mice.	20, 40 mg/kg/d	↑	—	—	—	ZO‐1, Occludin and Claudin‐1↑	—	Wei, Liu, et al. (2021)
Curcumin	Caco2	5 μM	↑	—	—	—	ZO‐1, Claudin‐1 and Claudin‐7↑	—	Wang et al. (2017)
	DSS‐induced colitis in male BALB/c mice	100 mg/kg/d	↑	—	↑	—	ZO‐1, Occludin and Claudin‐1↑	IL‐23/Th17	Wei, Wang, et al. (2021)

Abbreviations: “↑”, increased expression; “↓”, decreased expression; CTX, cyclophosphamide; DSS, dextran sulphate sodium; HFD, high‐fat diet; TNBS, 2,4,6‐trinito‐benzene‐sulfonic acid.

**TABLE 2:** Comprehensive comparison of Th17 and Treg cells.

Category	Th17 cells	Treg cells
Core function	Promote pro‐inflammatory responses, recruit neutrophils/monocytes, disrupt tissue barriers	Maintain immune tolerance, suppress excessive inflammation, facilitate tissue repair
Key transcription factors	RORγt (core), STAT3	FoxP3 (core), STAT5
Signature cytokines	Secreted: IL‐17A/F, IL‐21, IL‐22, TNF‐α, Receptors: IL‐23R, IL‐6R	Secreted: IL‐10, TGF‐β, IL‐35, Receptors: IL‐10Rα

### 4.1 Baicalin

Baicalin, a flavonoid extracted from the root of the herb called Scutellaria baicalensis, has a wide range of pharmacological activities, including immunomodulation and anti‐inflammation. In a rat model of colitis, baicalin was found to increase the expression of IL‐10 by increasing the number of Treg cells in the lamina propria of the colon, while decreasing the number of Th17 cells and the expression of IL‐17A in the colon, and then increased the expression levels of ZO‐1 and Occludin, as well as Muc2 protein in the mucosal layer, which may be associated with increased concentrations of butyric acid in the colon (Zhu et al. 2020).

### 4.2 Baicalein

Baicalein is an aglycone derivative from Baicalin, which has various effects such as anti‐inflammatory and antibacterial. In many mouse models, baicalein has been shown to increase intestinal epithelial TJs expression and maintain intestinal epithelial barrier function (Li et al. 2022). In another study, we found that baicalin can promote the activation of AhR and induce its transfer to the nucleus and increase the expression level of AhR's downstream Cytochrome P450 1A1 Enzyme (CYP1A1) to induce the differentiation of Treg cells and inhibit the differentiation of Th17 cells. Therefore, Baicalein has the potential to improve the intestinal barrier by activating AhR to restore Th17/Treg balance (Liu et al. 2020).

### 4.3 Kaempferol

Kaempferol (Kae) is a natural flavonoid compound that is widely found in edible vegetables, fruits, and herbs. Kae was reported to increase the expression of ZO‐1, Occludin, and Claudin‐1 in DSS‐induced colitis in mice, thereby improving intestinal permeability. Further studies showed that Kae increased IL‐10 expression, suggesting that Kae has the potential to regulate Treg cells. This conjecture can be further supported by another in vitro study, which showed that Kae could enhance the suppressive function of Treg cells by regulating the expression and phosphorylation of FOXP3 (Qu et al. 2021).

### 4.4 Poncirin

Poncirin, a natural bitter flavanone glycoside abundantly present in many species of citrus fruits, has various biological benefits such as anti‐oxidant, anti‐microbial, anti‐inflammatory, and anti‐cancer activities. Kang and Kim (2016) found that LBP significantly inhibited Th17 cell differentiation and RORγt expression in the lamina propria of the colon and increased Treg cell differentiation and Foxp3 expression in a TCBS‐induced mouse colitis model. It also increased the expression of ZO‐1, Occludin, and Claudin‐1 in the colon. These beneficial effects may be related to its inhibition of NF‐κB activation.

### 4.5 Hesperidin

Hesperidin, an important component of citrus flavonoids, is an important natural phenolic compound with potential therapeutic effects in a variety of diseases. Guo et al. (2019) explored the protective effect of hesperidin on the intestinal barrier in vivo and in vitro and found that hesperidin could enhance the intestinal integrity likely by activating the expression of the Nrf2 antioxidant pathway, increasing the level of IL‐10 and Treg cells in the colon, and then enhancing the expression of ZO‐1 and Occludin in mice.

### 4.6 Tangeretin

Tangeretin is a polymethoxy flavonoid widely found in citrus fruits. It has various biological activities, such as antioxidant, anti‐inflammatory, neuroprotective, and anti‐cancer. It was shown that in a mouse model of intestinal barrier dysfunction, cellulose could effectively increase the expression of Occcludin, Claudin‐1, and ZO‐1 by inhibiting Th17 cell differentiation, promoting Treg cell differentiation, decreasing RORγt, IL‐17 mRNA expression, and increasing Foxp3 and IL‐10 mRNA expression (Chen et al. 2021; Eun et al. 2017) Furthermore, it was also shown that a significant dose‐dependent inhibition of Notch1 mRNA levels was observed after cellulose treatment during Treg polarization, suggesting that cellulose treatment may promote Treg cell expression by inhibiting the Notch1/Jagged1 signaling pathway to increase Treg cells. These findings suggest that cellulose may improve the intestinal barrier by regulating the Th17/Treg cell balance through the NF‐κB and Notch1 pathways (Xu et al. 2019).

### 4.7 Alpinin

Alpinin, the major flavonol isolated from galangal, has been shown to reduce intestinal permeability as well as improve intestinal TJs (Miao et al. 2019; Tan and Zheng 2018). Lv et al. observed that Alpinin significantly alleviated colitis and restored Th17/Treg balance in mice. Kaolin (15 and 30 mg/kg) significantly upregulated IL‐10 and Foxp3 levels in the colons, and 30 mg/kg of kaolin also downregulated IL‐17 and RORγt levels. Another study further found that kaolin was a potential AhR activator that could directly promote Treg differentiation by inhibiting the methylation level of the Foxp3 promoter region, promoting CREB binding to the Foxp3 promoter region, and upregulating Foxp3 expression while slightly inhibiting Th17 differentiation (Lv et al. 2018). Thus, Alpinin has the potential to significantly improve colitis and thus the intestinal barrier in mice by restoring the Th17/Treg balance.

### 4.8 Naringenin

Naringenin is a naturally occurring major flavanone widely found in citrus fruits with a wide range of biological and pharmacological activities, including potential immunomodulatory functions. In Caco2 cells, naringenin was found to decrease intestinal permeability and increase the expression of some TJs proteins such as ZO‐1, ZO‐2, ocludin‐1, −3, and −4 (Noda et al. 2012). Similar to Alpinin, naringenin also acted as an AhR activator that significantly increased the proportion of Treg cells in mice (Wang et al. 2012). In contrast, the immunosuppressive activity of naringenin was attenuated by AhR antagonists, suggesting the potential of naringenin to improve the intestinal barrier by affecting Treg cells in an AhR‐dependent manner.

### 4.9 Phloretin

Phloretin is a flavonoid compound that is abundant in apples and many plants. It has a wide range of pharmacological effects, including anti‐inflammatory, antioxidant, and antibacterial effects. Many studies have shown that phloretin has the potential to improve intestinal barrier integrity by restoring disturbed Th17/Treg homeostasis through multiple pathways. Wu et al. (2019) found that phloretin restored Th17/Treg homeostasis and then increased intestinal integrity by inhibiting CD4^+^ IL‐17^+^ cells in the spleen, IL‐17A production in the colon, and increasing IL‐10 production in the colon of DSS‐induced colitis mice. Notably, this effect of phloretin was closely associated with the increased abundance of *Bacteroides*. In addition, another study showed that phloretin could inhibit the production of IL‐17A by the NF‐κB pathway as well as the upstream PPARγ pathway (Zhang et al. 2019). Similarly, a clinical study showed that phloretin inhibited Stat3 phosphorylation and promoted Stat5 phosphorylation during CD4^+^ T cell polarization, which in turn promotes T cell differentiation to Treg cells. In addition, phloretin could also promote AMPK phosphorylation in CD4^+^ T cells through the glycolytic AMPK signaling pathway while decreasing the level of mTOR phosphorylation (Jiao et al. 2020). These findings suggest that phloretin can regulate the Th17/Treg balance to improve the intestinal barrier by activating the NF‐κB, PPARγ, and AMPK pathways and increasing the abundance of *Bacteroides*.

### 4.10 Quercetin

Quercetin (QU) is the most abundant flavonoid compound found in fruits and vegetables. It has been reported to directly modulate TJP to enhance intestinal barrier function. The Sprague–Dawley (SD) rats that were supplemented with QU showed lower plasma DAO, D‐lactate, and endotoxin levels, and higher expression of TJs protein ZO‐1, claudin 1, and Occludin (Zheng et al. 2018). This may be associated with the reduced expression of IL‐17A, suggesting that QU may positively affect intestinal barrier function by influencing cytokines produced by Th17 cells. Further studies supported this conjecture. QU was able to repress the transcriptional activity of STAT3 by activating PPAR and redistributing the co‐repressor SMRT from PPAR to STAT3, which in turn inhibited Th17 cell differentiation (Yang, Shi, et al. 2022). The above findings suggest that QU has the potential to activate the PPAR signaling pathway to regulate Th17/Treg balance and improve intestinal barrier integrity. In addition, QU is also a natural AhR agonist. Yuan et al. reported that QU restored colonic FoxP3 expression in DSS‐induced colitis, while reducing the number of Th17 cells and RORγt expression, which in turn reduced intestinal permeability as well as upregulated the expression he Claudin‐1. In contrast, these effects of QU were significantly inhibited in Ahr^−/−^ mice (Riemschneider et al. 2021). These results further suggest that quercetin can also regulate Th17/Treg balance and improve the intestinal barrier through activation of AhR.

### 4.11 Apigenin

Apigenin is a flavonoid with various pharmacological activities and low toxicity. It is widely found in many fruits and vegetables, especially celery and herbs, and has anti‐obesity, anti‐inflammatory, and anti‐cancer properties. It has been reported that in HFD mice, apigenin significantly improved the expression of intestinal TJs protein ZO‐1, occludin, which in turn improved intestinal permeability. At the same time, apigenin also increased the expression of IL‐10, suggesting that apigenin may have the potential to regulate Treg cells (Qiao et al. 2021).

### 4.12 (‐)‐Epigallocatechin‐3‐Gallate

Phenolic acids are naturally occurring compounds that are abundant in plants, especially in fruit skins and seeds. Epigallocatechin‐3‐gallate (EGCG), a polyphenol derived from tea, is known to have antibacterial, anti‐inflammatory, anti‐tumor, antioxidant, and anti‐aging effects. Many in vitro and in vivo studies have demonstrated that EGCG was able to protect the intestinal barrier. Ran et al. found that EGCG improved cyclophosphamide‐induced intestinal permeability damage and increased the expression of IL‐10. In vivo, EGCG not only upregulated Foxp3 expression and inhibited RORγt levels, but also restored Th17/Treg balance by inhibiting HIF‐1α and p‐STAT3, which in turn inhibited Th17 differentiation and increased Treg cell production (Wei, Liu, et al. 2021).

### 4.13 Curcumin

Curcumin is a natural phenolic substance extracted from the rhizome of the plant turmeric and has long been used as a traditional herbal medicine in China and Southeast Asia. It has anti‐inflammatory, anti‐infective, anti‐coagulant, and immunomodulatory properties and protects the intestinal mucosa and repairs intestinal tissues. In vivo *and* in vitro studies have shown that curcumin significantly improved intestinal integrity. The TJs proteins such as Ocludin, ZO‐1, and claudin‐3 were upregulated in C57BL/6J mice that were fed with curcumin (Wang et al. 2017). In addition, it was also reported to regulate Th17/Treg balance by inhibiting the IL‐23/Th17 pathway, downregulating IL‐6, IL‐17, IL‐23, and upregulating the anti‐inflammatory cytokine IL‐10 (Wei, Wang, et al. 2021).

### 4.14 Resveratrol

Resveratrol is a natural plant polyphenol that is widely found in grapes, thuja, berries, and peanuts and has a variety of pharmacological effects such as anti‐oxidant, anti‐aging, anti‐inflammatory, anti‐cancer, anti‐obesity, anti‐diabetic, cardioprotective, and neuroprotective. Resveratrol also has several intestinal bioactivities. Studies have shown that resveratrol treatment significantly restored the reduction of TJs proteins, especially Occludin, caused by cyclophosphamide (Song et al. 2022). In addition, resveratrol can affect intestinal immune cells and lymphoid tissue. In colitis mice, resveratrol treatment increased the number of Treg cells as well as IL‐10 and downregulated the number of Th17 cells in the MLN (Yao et al. 2015). It is suggested that resveratrol has the potential to improve the intestinal barrier by modulating the Th17/Treg balance.

## 5 Toxicity and Safety Research

In recent years, polyphenols have received considerable attention due to their potential health benefits. However, there are also growing concerns about their potential toxicity. The safety of polyphenols depends on a variety of factors, including the type of polyphenol, the dose, and the duration of exposure. Most studies have shown that polyphenols are safe to consume as part of a balanced diet and no side effects have been observed. However, when consumed in large amounts, polyphenols can cause liver damage, kidney damage, and even death. QU is a safe dietary supplement in the dose range of 62–250 mg/kg in rats or 12.5–50 mg/kg in mice (Cunningham et al. 2022). However, at higher doses, it has been shown to cause liver and kidney damage in rats (Granato et al. 2017). Similarly, higher doses of EGCG at 2500 mg/kg have been shown to cause liver damage (Mazzanti et al. 2009). The potential toxicity of resveratrol has also been studied. Several studies suggested that high doses of RSV may be toxic to cells and tissues (Khan et al. 2016). Resveratrol is nephrotoxic and a dose of 3000 mg/kg RSV could cause significant increases in the blood urea nitrogen (BUN) and creatinine levels, as well as the liver enzymes (Crowell et al. 2004). More importantly, RSV acts as a pro‐oxidant in increasing oxidative stress while promoting DNA damage at higher doses. In a mouse model of indomethacin‐induced gastric ulcers, low doses (2 mg/kg) of resveratrol can increase PGE2 levels; PGE2 has a stomach‐protective effect, thus acting as a protective agent for the gastric mucosa, while high doses (10 mg/kg) of resveratrol inhibit PGE2 synthesis, further exacerbating gastric ulcers (Dey et al. 2009). In addition, some studies have shown that high doses of polyphenols can cause gastrointestinal problems, such as diarrhea, nausea, and abdominal pain. Other polyphenols, such as hesperidin and curcumin, have been shown to be safe at recommended doses, but their potential toxicity at high doses still requires further research. Further research is needed to determine the optimal dosage and safety of polyphenols to improve health outcomes.

## 6 Conclusion and Future Prospects

Impaired intestinal barrier function is a pathophysiological hallmark of multiple disorders, including IBD, IBS, and type 1 diabetes mellitus, underscoring its significance as a therapeutic target. Emerging evidence indicates that dietary polyphenols enhance intestinal barrier integrity by modulating the Th17/Treg immune axis and upregulating TJ proteins. Preclinical studies demonstrate that these compounds suppress pro‐inflammatory Th17 responses while promoting Treg‐mediated immune tolerance. However, clinical translation remains constrained by overreliance on animal and in vitro models, insufficient validation in human trials, and unresolved causality between Th17/Treg balance restoration and barrier repair. To address these limitations, future research should prioritize the following directions: First, elucidating microbiome‐polyphenol interactions, particularly how gut microbiota‐derived metabolites (e.g., urolithins from ellagitannins) influence immune‐barrier crosstalk. Second, engineering advanced delivery systems, such as nanoencapsulated polyphenols or polyphenol‐drug conjugates, to optimize bioavailability and target‐specific efficacy. Third, employing multi‐omics approaches (e.g., single‐cell RNA sequencing, metabolomics) to mechanistically validate polyphenol‐driven pathways (e.g., IL‐10/STAT3 signaling) in barrier restoration. Fourth, conducting phased clinical trials, beginning with pharmacokinetic profiling anddose‐response studies, followed by multicenter randomized controlled trials (RCTs), to establish biomarker‐guided protocols (e.g., serum zonulin, fecal calprotectin) for polyphenol interventions. Additionally, combinatorial strategies (e.g., anthocyanins with EGCG) warrant exploration to amplify synergistic effects on Th17/Treg equilibrium and environmental triggers (e.g., gluten in celiac disease). Interdisciplinary collaboration integrating immunology, bioengineering, and precision nutrition will be critical to transform polyphenols from dietary components into evidence‐based adjuvants for intestinal barrier‐related pathologies.

## Author Contributions

**Jiaojiao Fu:** writing – original draft (equal). **Sijing Liu:** writing – review and editing (equal). **Zhenghua Zhao:** writing – original draft (equal). **Da Zhou:** writing – review and editing (equal).

## Conflicts of Interest

The authors declare no conflicts of interest.

## Data Availability

No data were used for the research described in the article.
